# Non-coding RNAs predict recurrence-free survival of patients with hypoxic tumours

**DOI:** 10.1038/s41598-017-18462-z

**Published:** 2018-01-09

**Authors:** Victor D. Martinez, Natalie S. Firmino, Erin A. Marshall, Kevin W. Ng, Brennan J. Wadsworth, Christine Anderson, Wan L. Lam, Kevin L. Bennewith

**Affiliations:** Department of Integrative Oncology, British Columbia Cancer Agency, Vancouver, B.C V5Z 1L3 Canada

## Abstract

Hypoxia promotes tumour aggressiveness and reduces patient survival. A spectrum of poor outcome among patients with hypoxic tumours suggests that additional factors modulate how tumours respond to hypoxia. PIWI-interacting RNAs (piRNAs) are small non-coding RNAs with a pivotal role in genomic stability and epigenetic regulation of gene expression. We reported that cancer type-specific piRNA signatures vary among patients. However, remarkably homogenous piRNA profiles are detected across patients with renal cell carcinoma, a cancer characterized by constitutive upregulation of hypoxia-related signaling induced by common mutation or loss of von Hippel-Lindau factor (VHL). By investigating >3000 piRNA transcriptomes in hypoxic and non-hypoxic tumors from seven organs, we discovered 40 hypoxia-regulated piRNAs and validated this in cells cultured under hypoxia. Moreover, a subset of these hypoxia-regulated piRNAs are regulated by VHL/HIF signaling *in vitro*. A hypoxia-regulated piRNA-based score (PiSco) was associated with poor RFS for hypoxic tumours, particularly Stage I lung adenocarcinomas, suggesting that hypoxia-regulated piRNA expression can predict tumour recurrence even in early-stage tumours and thus may be of clinical utility.

## Introduction

Many solid tumours develop poorly-oxygenated (hypoxic) regions. Hypoxic conditions are known to upregulate >90 genes associated with anaerobic glycolysis, pH regulation, angiogenesis, cellular migration, and metastasis^1,2^ through the activity of the heterodimeric transcription factors hypoxia-inducible factor-1 (HIF-1) and HIF-2^3^. Hypoxic tumour cells increase the risk of metastatic disease and reduce the effectiveness of radiation therapy and most forms of chemotherapy, resulting in an aggressive tumour phenotype and decreased patient survival^4–6^. There are several methods to identify hypoxic tumours, and patients with hypoxic tumors typically have worse outcome than patients with normoxic tumors (e.g. lung tumours^7–9^). However, there is a spectrum of poor outcomes in patients with hypoxic tumors, and an important unmet need in the oncology field is to identify those patients that will have the worst outcome.

Hypoxia has a broad effect on miRNA expression. It can disrupt its biogenesis and induce increase in expression of specific miRNAs, such as miR-210^10–12^. However, its effect on other small non-coding RNAs (sncRNAs), such as PIWI-interacting RNAs (piRNAs) are unexplored. PiRNAs are abundantly expressed in germline cells, regulating genomic stability by recognizing DNA target sequence, and participating in the recruitment of the necessary machinery to induce epigenetic silencing of transposable elements^13–16^. Recent evidence indicates that they are expressed and functionally active in somatic tissue and also linked to epigenetic mechanisms of cancer development^17–28^. Moreover, we and others have recently identified distinct, cancer-type specific piRNA expression patterns across many tumour types^18,29–39^.

Although little is known about how piRNA expression is regulated in somatic cells or solid tumours, our data from patients with renal cell carcinoma (RCC) suggest a role for the solid tumour microenvironment in regulating piRNA expression and perhaps contributing to the heterogeneous piRNA expression observed between patients^18^. We found that piRNA expression was remarkably consistent between RCC tumours, a tumour type that commonly harbour loss-of-function mutations in von Hippel-Lindau factor (VHL), causing constitutive, oxygen-independent stabilization of HIF-1α^3^ and HIF-mediated upregulation of hypoxia-associated gene products. We therefore postulated that piRNA expression may be affected by hypoxia in non-RCC tumours and that piRNAs associated with hypoxia may identify patients at higher risk of recurrence, thus facilitating the development of piRNA signatures for patient prognosis.

**Figure 1:**
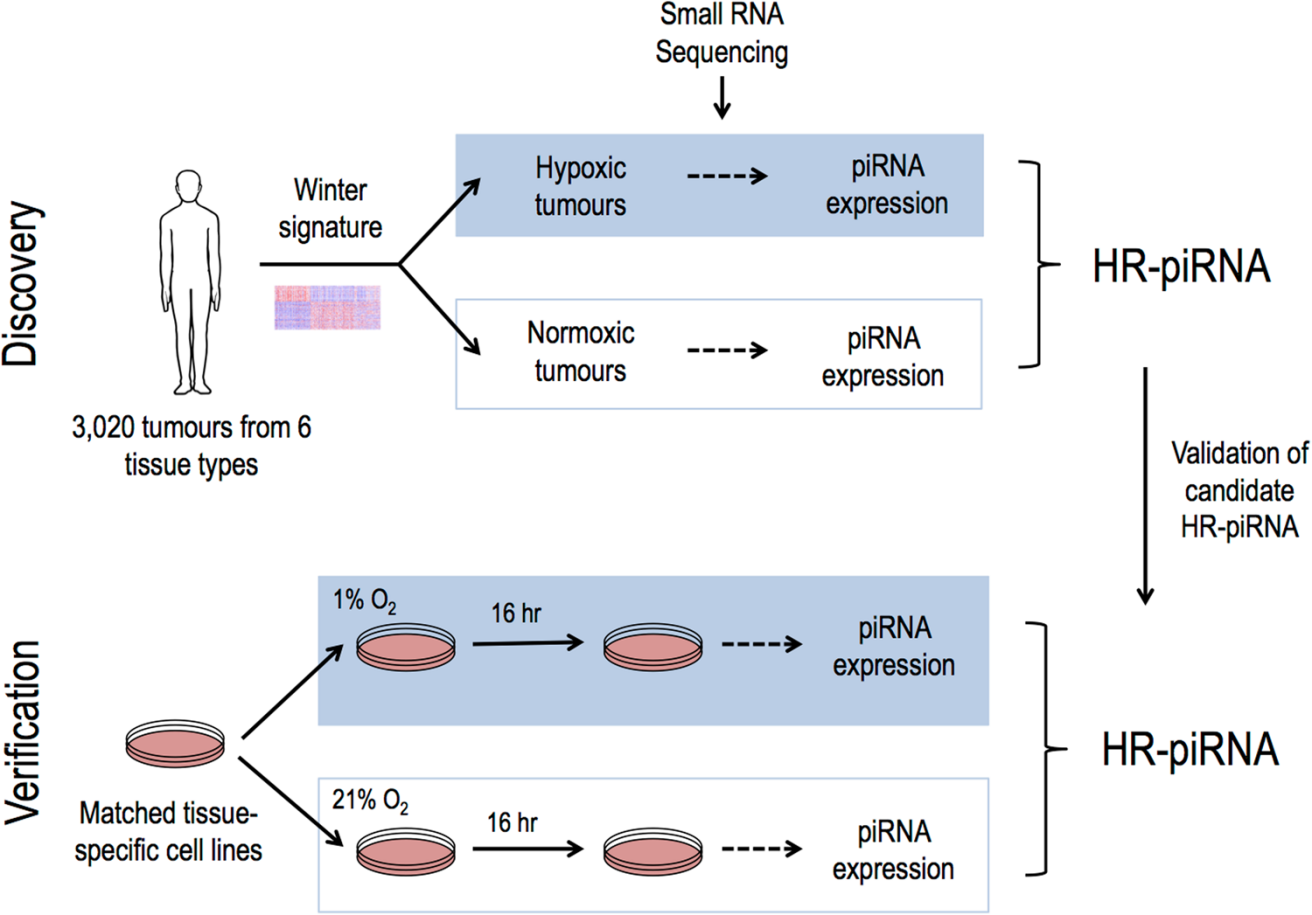
Experimental design for discovery and validation of hypoxia-regulated piRNAs. Hypoxic status from 3,020 human tumors was derived using an established metagene signature^41^. Expression of 23,440 human piRNAs was assessed using our previously published small RNA sequence data analysis pipeline. Hypoxia-regulated piRNAs were determined by comparing hypoxic and non-hypoxic tumors in a per tissue basis. Candidate hypoxia-regulated piRNAS derived from these analyses were further validated by *in-vitro* studies, using cell lines matching the tissues using during the discovery stage. These validated hypoxia-regulated piRNAs were considered for downstream analyses.

Using our custom small RNA sequence analysis pipeline^18,39,40^ we assessed 3,020 piRNA transcriptomes in hypoxic and non-hypoxic patient tumours derived from seven different organs (Fig. 1). We discovered that hypoxia is associated with increased expression of a subgroup of piRNAs in patient tumours, and that most of these piRNAs are also overexpressed in tumour cell lines exposed to hypoxic conditions or VHL disruption *in vitro*. Remarkably, we have also found that that the expression of these hypoxia-regulated piRNAs (hypoxia-regulated piRNAs) can be used to predict recurrence-free survival (RFS) of patients with hypoxic tumours.

## Methods

### Experimental design and identification of hypoxic status in tumours

To determine if hypoxia can induce changes in piRNA expression, a total of 3,020 tumour samples processed by The Cancer Genome Atlas (TCGA) Research Network were analyzed for hypoxic status based on expression of 143 genes included in the Winter hypoxia metagene signature^41^. Consensus clustering analyses were performed for each tumour type^42^ and the resulting tumour clusters were then compared with the Winter hypoxia metagene signature. The cluster with the most concordant gene expression signature was classified as “hypoxic”, while the most discordant was defined as “non-hypoxic”. Samples that did not match the Winter signature were defined as “unclassified”.

### Cell lines exposed to hypoxia ***in vitro***

We used 9 tumour cell lines that resembled the TCGA tumour types analyzed above. Selected cell lines were: A549 (lung), FaDu (pharynx), HCC15 (lung), HCC4006 (lung), Mia-Paca-2 (pancreas), NCI-H841 (lung), OE33 (esophagus), PANC1 (pancreas), and SiHa (cervix). Cell lines were obtained from ATCC and passaged using 0.1% trypsin citrate buffer + EDTA. The culture media used was: Dulbecco’s Modified Eagle Medium supplemented with 10% Fetal-Bovine Serum (FBS) for PANC1 and Mia-Paca-2; Dulbecco’s Modified Eagle Medium: F12 with 10% FBS for NCI-H841; RPMI1640 + 10% FBS for HCC15 and HCC4006; RPMI-1640 + 2 mM glutamine + 10% FBS for OE33; F12K with 10% FBS for A549; Eagle’s MEM + 10% FBS for FaDu; MEM + 10% FBS for SiHa.

For hypoxia exposure, 70–80% confluent plates were transferred to a controlled-atmosphere hypoxia chamber and incubated for 16 hours at 1% O_2_. Cells were then rinsed with cold PBS, scraped off the plate in 1 mL of PBS into a sealed eppendorf tube, and taken out of the hypoxia chamber on ice. After spinning the cells at 1700 rpm for 5 min and aspirating PBS, cells were lysed in 1 mL of Trizol.

### RNA extraction for Sequencing

Total RNA was extracted using TRIzol reagent according to the manufacturer’s instructions, and eluted in RNase-free water. RNA concentration and quality were determined using a NanoDrop™ 2100 spectrophotometer, and samples were stored at −70 °C. RNA was cleaned up as needed using a Zymogen kit. The same sequencing and analysis protocols were applied to TCGA tumours and tumour cell lines^43^.

### Small RNA sequencing

Small RNA sequencing libraries were generated at Canada’s Michael Smith Genome Sciences Centre and sequenced using the Illumina Genome Analyzer and HiSeq. 2000 platform, for both TCGA tumours and cell lines. We extracted unaligned FASTQ reads from BAM files of each tumour from the TCGA cohort, which were retrieved from the cgHub data repository (dbgap Project ID: 6208) using the PartekFlow™ platform. To make data analysis protocols comparable, we applied the same procedure for the hypoxic/normoxic cell lines sequenced. Reads were subjected to quality control in order to exclude non-biological reads, such as 3′ adapter sequences. To avoid overlapping with miRNA-derived reads, a secondary filtering step was applied based on read trimming, retaining reads that were ≥23 bp with phred scores ≥20.

### Generation of piRNA transcriptomes

For each tumour cell line analyzed, filtered reads were aligned to the human genome (GRCh37/hg19) using the STAR aligner^44^, with specific parameters for short reads: i) only reads 23 bp or longer matched to the genome, ii) number of mismatches < = 5% of mapped length, and iii) splicing switched off. Mapped reads were subsequently quantified within the PartekFlow™ platform using a piRNA-specific annotation file generated by the piRNABank (http://pirnabank.ibab.ac.in/)^45^. This reference transcriptome considers widely accepted piRNA sequence features, such as sequence bias for uracil in the 1st and adenine in the 10th position, although known biases for 2-o-methylation in the 3′ end were not considered.

### VHL knockdown by siRNA

A549 cells were treated with siRNA to VHL at 5 nM using Dharmafect for 48 hrs at 21% O_2_ (Ambion, Inc. Cat#(Lot): non-targeting control #4390843, siRNA 1 VHL #4390824(s14789), siRNA 2 VHL #4392420(s14790), siRNA 3 VHL #4392420(s14791)). RNA was extracted using Quick-RNA MiniPrep Kit column purification (Zymo Research, #R1055), and resuspended in nuclease-free water. cDNA conversion was performed on 20 ng of isolated RNA using the TaqMan (R) MicroRNA Reverse Transcription Kit (Thermo Fisher, #4366596) and custom reverse transcription primers for DQ580854 (context sequence: TGAGGAGCCAATGGGGCGAAGCTACCATC, target sequence: UGAGGAGCCAAUGGGGCGAAGCUACCAUC), DQ590404 (context sequence: TGGTGTATGTGCTTGGCTGAGGAGCCAATGG, target sequence: UGGUGUAUGUGCUUGGCUGAGGAGCCAAUGG), and DQ596992 (context sequence: GCAATAACAGGTCTGTGATGCCCTTAGA, target sequence: GCAAUAACAGGUCUGUGAUGCCCUUAGA). All primers used FAM as a reporter dye and NFQ as a reporter quencher. RT-qPCR using paired primers was performed for 40 cycles, and RQ values were calculated with the ddCt method, with each treatment normalized to its respective endogenous U6 control. Experiments were performed in biological duplicates.

### Survival Analysis

TCGA samples with complete recurrence-free survival and piRNA expression data (RPKM) from different tumour types (HNSC, LUAD, and LUSC) were included in the Cox proportional hazard (COXPH) model. Only hypoxia-regulated piRNAs that were validated in hypoxic vs normoxic cell lines of the same cell type were considered for the generation of predictive signatures. Age at diagnosis and clinical stage were included as know stratification factors in the COXPH model for each tumour type, in addition to smoking status and history for LUAD and LUSC patients, but removed in the score evaluation. PiRNAs were included in the piRNA score (PiSco) if they were found to contribute significantly to the classification of patient survival by the COXPH model (p-value ≤0.1), and the optimized model with the lowest p-value was used for further analysis. PiSco was generated by multiplying the expression value of a given piRNA by its hazard coefficient, then summing the transformed gene expression values per sample^39,46^. Risk scores were then ranked and divided into tertiles, selecting upper and lower tertiles to compare using the log-ranked method with a significance threshold of p ≤ 0.05. The recurrence-free survival of the high-score cohort was then compared to that of the low-score tertile.

## Results

### Identification of hypoxic status in human tumours

We used the metagene signature previously established by Winter *et al*. containing 143 transcripts associated with tumour hypoxia (92 up-regulated and 51 down-regulated; Table S1) for a consensus clustering analysis to stratify TCGA tumour samples into “hypoxic”, “non-hypoxic”, or “unclassified” tumour groups (Figure S1A–H)^41,42^. In total, 3,020 human tumours derived from cervix (CESC, n = 306), head and neck (HNSC, n = 522), esophagus (ESCA, n = 196), lung adenocarcinoma (LUAD, n = 517), pancreas (PAAD, n = 179), lung squamous (LUSC, n = 501), kidney (KIRC, n = 534), and ovary (OVCA, n = 265) were used for our analyses (Table 1). Each tissue type was analyzed individually. Most tumours were clearly classified as hypoxic or non-hypoxic in concordance with the metagene signature, and we therefore focused on these tumours for subsequent discovery of hypoxia-associated piRNA expression. Kidney and ovarian tumours could not be clearly classified as hypoxic or non-hypoxic based on the Winter signature (Figure S1G–H), and were therefore not included for further analysis.

**Table 1:** Hypoxic and non-hypoxic human tumours.

Tumour type	Total number of tumours analyzed	Number of tumours in the hypoxic cluster	Number of tumours in the non-hypoxic cluster
Cervical	306	102	130
Esophageal	196	83	97
Head and neck	522	215	178
Kidney	534	n/a	n/a
Lung (LUAD*)	517	235	198
Lung (LUSC*)	501	212	219
Ovarian	265	n/a	n/a
Pancreatic	179	85	72
TOTAL	**3,020**	**932**	**894**

Clustering of human tumours by tissue type into hypoxic and non-hypoxic clusters according to the Winter metagene signature^41^. *LUAD = Adenocarcinomas, LUSC = Squamous cell carcinomas.

### piRNA expression is selectively deregulated by hypoxia in human tumours

We then sought to identify differences in piRNA transcriptomes between hypoxic and non-hypoxic tumours. Expression levels of 23,440 human piRNAs were determined on a per tumour basis (Figure S2) using our previously published custom small RNA sequencing analysis pipeline^18^. To identify the most robust changes in piRNA expression between hypoxic and non-hypoxic groups, piRNAs were only included in analyses if they had a median expression ≥10 RPKM in at least one of the groups (hypoxic and/or normoxic) and a minimum of 2-fold change in median RPKM expression values. Comparisons were performed using the nonparametric Mann-Whitney U test, with a false discovery rate correction for multiple testing using the Benjamini-Hochberg method^47^.

**Figure 2:**
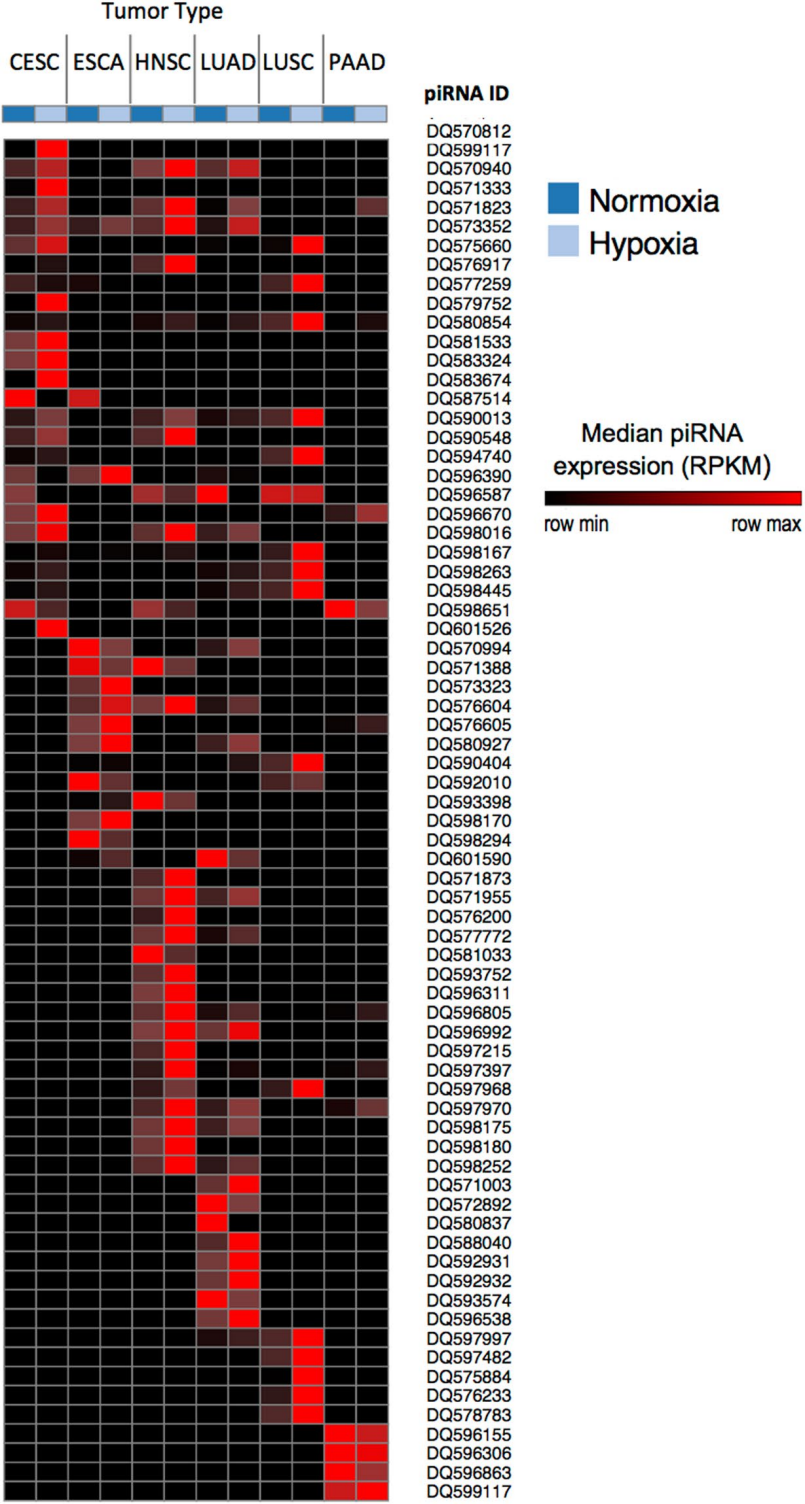
Hypoxia-regulated piRNAs in human tumours. Heatmap representing the median RPKM expression values of 71 robustly expressed piRNAs (a median expression ≥10 RPKM in either hypoxic or normoxic tumors, and a minimum of 2-fold variation in median RPKM expression values) across different tissue types. Expression of each piRNA is shown for normoxic (green) and hypoxic (blue) tumors on a per tissue basis. Represented tissue types are: cervical squamous cell carcinoma (CESC), esophageal carcinoma (ESCA), head and neck squamous cell carcinoma (HNSC), lung adenocarcinoma (LUAD), lung squamous cell carcinoma (LUSC) and pancreatic adenocarcinoma (PAAD).

A total of 71 unique piRNA sequences showed significant differences in expression between hypoxic and non-hypoxic tumours (Table S2, Fig. 2). Of these hypoxia-regulated piRNAs 33 (46.5%) were deregulated in two or more tumour types, while the other 38 (53.5%) showed a tumour type-specific response to hypoxia (Table 2, Figure S3). HNSC, LUAD and CESC were the tumour types with the greatest number of hypoxia-regulated piRNAs (30, 33, and 26, respectively) compared to ESCA, LUSC, and PAAD (17, 17, and 12, respectively).

**Table 2:** Shared and common expression of hypoxia regulated piRNAs.

Tissue types	piRNAs per group	piRNA IDs
ESCA	3	DQ573323, DQ598170, DQ598294
LUSC	4	DQ578783, DQ576233, DQ575884, DQ597482
PAAD	4	DQ599117, DQ596306, DQ596155, DQ596863
CESC	7	DQ601526, DQ583674, DQ571333, DQ579752, DQ570812, DQ583324, DQ581533
HNSC	7	DQ581033, DQ598180, DQ593752, DQ576200, DQ596311, DQ571873, DQ597215
LUAD	8	DQ580837, DQ588040, DQ592932, DQ596538, DQ571003, DQ572892, DQ593574, DQ592931
CESC/ESCA	1	DQ587514
CESC/LUSC	1	DQ594740
CESC/PAAD	1	DQ596670
ESCA/LUSC	1	DQ592010
ESCA/PAAD	1	DQ576605
HNSC/LUSC	1	DQ597968
LUAD/LUSC	1	DQ597997
CESC/HNSC	2	DQ590548, DQ576917
ESCA/HNSC	2	DQ593398, DQ571388
ESCA/LUAD	3	DQ601590, DQ580927, DQ570994
HNSC/LUAD	5	DQ596992, DQ598175, DQ571955, DQ598252, DQ577772
CESC/ESCA/LUAD	1	DQ596390
CESC/ESCA/LUSC	1	DQ577259
CESC/HNSC/PAAD	1	DQ598651
ESCA/HNSC/LUAD	1	DQ576604
ESCA/LUAD/LUSC	1	DQ590404
CESC/HNSC/LUAD	2	DQ598016, DQ570940
CESC/LUAD/LUSC	3	DQ575660, DQ598263, DQ598445
HNSC/LUAD/PAAD	3	DQ596805, DQ597397, DQ597970
CESC/ESCA/HNSC/LUAD	1	DQ573352
CESC/ESCA/HNSC/LUSC	1	DQ598167
CESC/HNSC/LUAD/PAAD	1	DQ571823
CESC/HNSC/LUAD/LUSC	2	DQ590013, DQ596587
CESC/HNSC/LUAD/LUSC/PAAD	1	DQ580854

The number of piRNAs for a single tumour type or combinations indicate those piRNAs that are hypoxia regulated exclusively in that or those tissues (and not the total number of hypoxia-regulated piRNAs in the tissue).

### ***In vitro*** tumour models recapitulate hypoxia-regulated piRNA expression patterns

**Figure 3:**
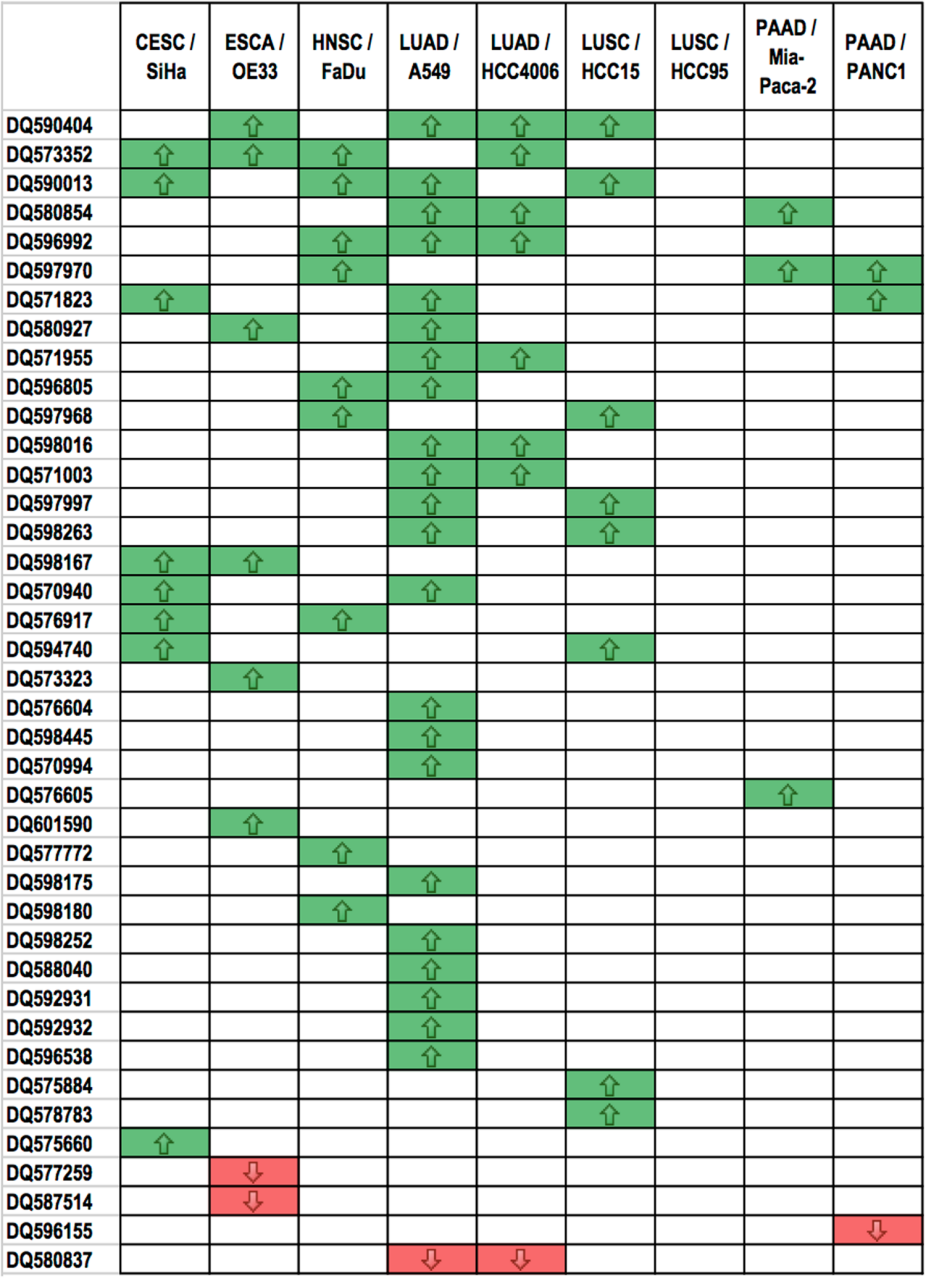
Validated hypoxia-regulated piRNAs in human tumour cell lines. List of hypoxia-regulated piRNAs identified in the TCGA analyses that are also significantly upregulated (green) or downregulated (red) in hypoxic tumour cells compared to normoxic tumour cells from the same tissue type. The piRNAs are ordered from piRNAs upregulated in the highest number of cell lines tested (top) to downregulated in the largest number of cell lines (bottom).

To validate whether changes in hypoxia-regulated piRNA expression observed in clinical samples are driven by hypoxia, piRNA expression levels were examined in a panel of nine cell lines from the corresponding tumour types used in the previous analysis, following exposure to hypoxic (1% O_2_ for 16 hours) or normoxic (21% O_2_) conditions (Fig. 1). Forty out of the 71 hypoxia-regulated piRNAs identified in hypoxic clinical tumours showed hypoxic regulation in the associated tumour cell lines. Of these validated hypoxia-regulated piRNAs, 36 were upregulated and 4 downregulated in hypoxic cells (Fig. 3).

### von Hippel-Lindau factor (VHL)/HIF signaling axis regulates the expression of hypoxia inducible piRNAs

**Figure 4:**
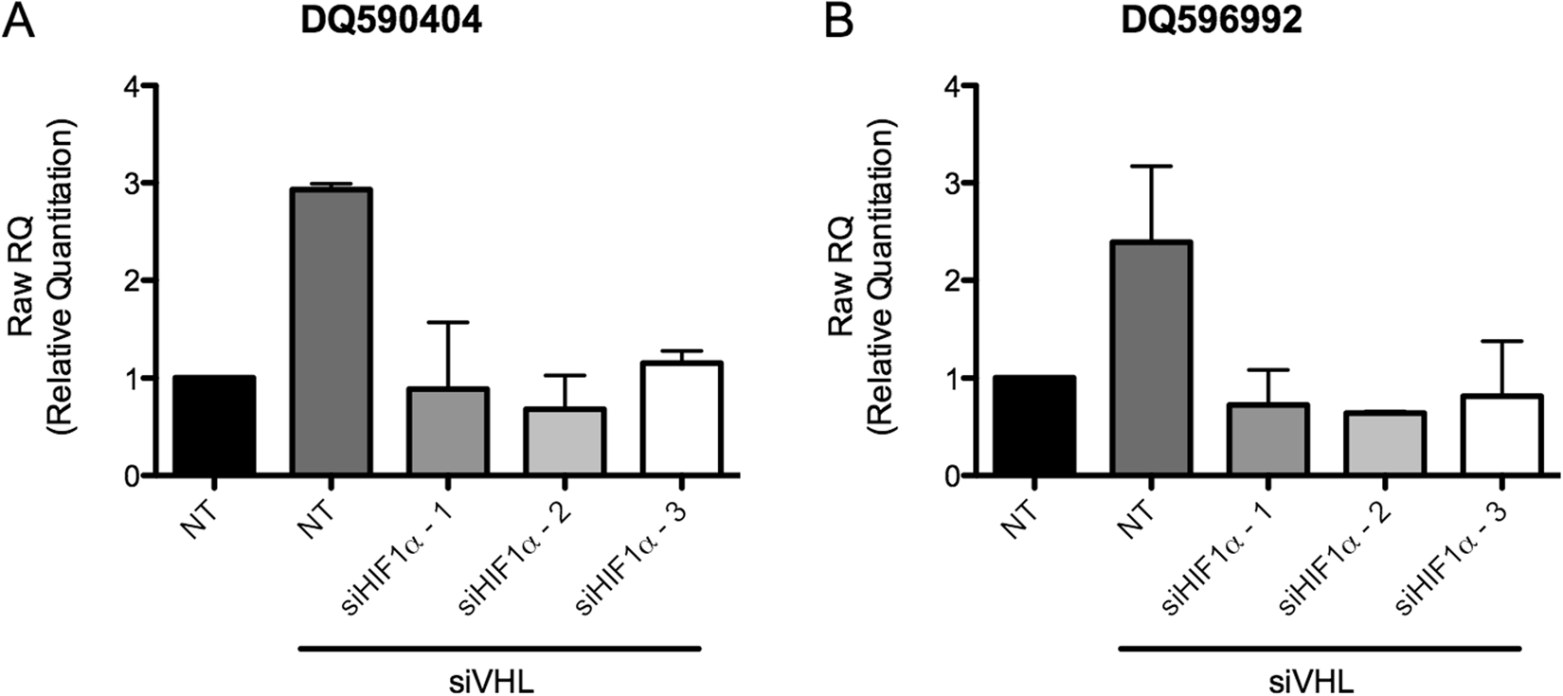
Silencing of VHL induces expression of HIF-1α and increases expression of hypoxia-regulated piRNAs. A549 cells were treated for 48 hours with non-targeting siRNA (NT), siRNA targeting VHL, or siRNA targeting VHL and three siRNA constructs targeting HIF-1α. (**A**) DQ590404 and (**B**) DQ596992 piRNA expression is increased by VHL knockdown in a HIF-1α dependent manner.

We previously observed remarkable homogeneneity in piRNA expression in RCC tumors^18^, which are known to have a high frequency of loss-of-function mutations in the *VHL* gene that causes constitutive stabilization of HIF-1α. We hypothesized that the induction of those piRNAs upregulated by exposure to 1% O_2_ would be dependent on HIF-1α. To address this hypothesis we selected A549 lung adenocarcinoma cells (with the highest number of hypoxia-regulated piRNAs detectable at the sequencing level) to test the effect of siRNA-mediated knockdown of VHL on expression of DQ590404 and DQ596992, which were two of the most highly overexpressed hypoxia-regulated piRNAs in A549 cells. We found that siRNA-mediated knockdown of VHL increased expression of both piRNAs (Fig. 4A,B), and that this induction was inhibited when VHL knockdown was combined with siRNA-mediated knockdown of HIF-1α, indicating that HIF-1α stabilization induced by VHL knockdown was responsible for the increase in piRNA expression.

### A hypoxia-regulated piRNA signature delineates risk of recurrence in patients with hypoxic tumours

**Figure 5:**
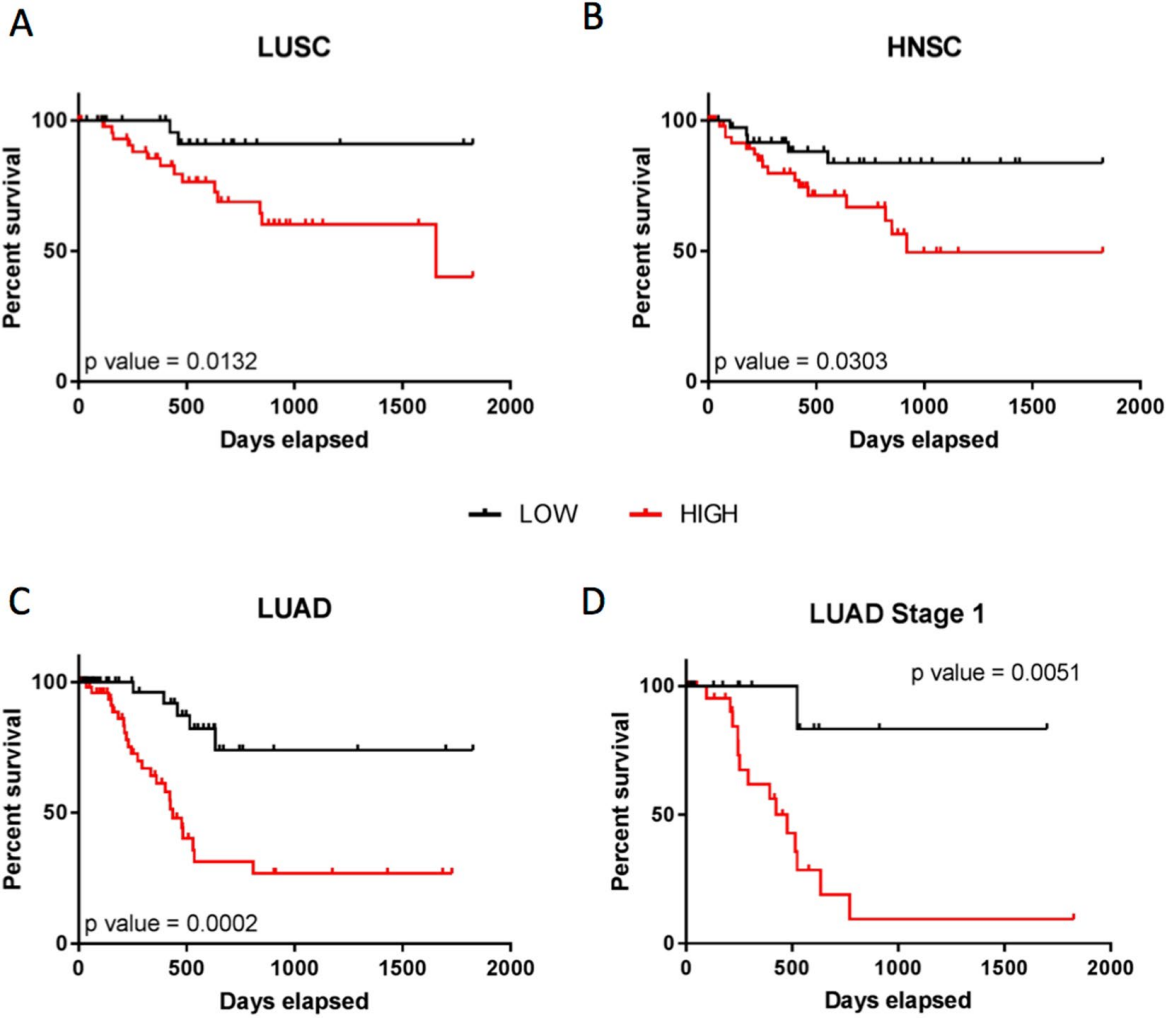
PiSco predicts recurrence-free survival (RFS) of patients with hypoxic tumours. The tumour-type specific score of piRNAs upregulated in hypoxia (PiSco) can classify patients with hypoxic tumours according to the Winter metagene signature into low and high RFS (**A**) LUSC, (**B**) HNSC, (**C**) LUAD). The LUAD PiSco is also a strong predictor of RFS in patients with hypoxic stage I LUAD (**D**).

Considering the adverse effects of tumour hypoxia in tumour recurrence, we next investigated if a hypoxia-regulated piRNA expression signature was associated with recurrence-free survival (RFS). We first restricted our analyses to patients with hypoxic tumours as defined by the Winter hypoxia metagene signature. Using a Cox proportional hazard (COXPH) model, we developed a piRNA-based score (PiSco) for each tumour type by weighting the expression levels of hypoxia-regulated piRNAs in the tumour type using the obtained Cox coefficients and summing the resulting values, as previously described^48^. Weighted contribution of piRNAs to tissue-specific PiSco signature is shown in Table 3. After correction by known cancer-specific risk factors (e.g., age, stage), comparison of the upper and lower PiSco tertiles revealed that the combined expression of these hypoxia-regulated piRNAs could classify patients with hypoxic HNSC, LUSC, and LUAD tumours as low or high risk of recurrence-free survival (Fig. 5A–C).

**Table 3:** piRNA and expression values used to calculate PiSco, by tissue type.

	piRNA	Weighted Contribution to PiSco
LUAD	DQ590013	−2.74E-05
	DQ580854	−1.25E-03
	DQ571823	−1.59E-03
	DQ580927	1.11E-03
	DQ571955	2.10E-03
	DQ596805	2.97E-03
	DQ598016	−1.46E-03
	DQ597997	3.02E-03
	DQ570940	6.57E-05
	DQ570994	−2.96E-03
	DQ598175	1.49E-04
	DQ598252	5.99E-05
LUSC	DQ597997	−2.56E-03
	DQ598263	7.99E-06
	DQ594740	−1.22E-04
	DQ578783	3.26E-05
HNSC	DQ573352	2.08E-03
	DQ576917	−1.35E-03
	DQ577772	1.34E-03
CESC	DQ573352	−1.68E-02
	DQ590013	6.40E-05
	DQ598167	−5.42E-03
	DQ570940	1.80E-04
	DQ594740	−3.79E-04
	DQ575660	−3.13E-03
PAAD	DQ580854	7.17E-04
	DQ576605	1.37E-02
ESCA	No significant contribution of piRNAs	

Each piRNA that significantly contributes to tissue-specific survival classification is included in score, and scores are weighted based on coefficients from each Cox proportional hazards model.

To verify that the association between PiSco and RFS was not driven exclusively by late-stage tumours in the cohorts, we applied the same analysis strategy now only considering hypoxic Stage I tumours. Since the lung cancer cohort exhibited the largest number of Stage I cases (54 and 61 for LUSC and LUAD, respectively), we restricted these analyses to this tumour type. While results from LUSC showed a tendency to significance (p value = 0.0985), PiSco was able to appropriately classify early-stage LUAD patients into low or high risk groups for recurrence-free survival (p value = 0.0051, Fig. 5D).

## Discussion

Hypoxic tumours are known to be aggressive and resistant to radiotherapy and chemotherapy^6^. However, there is a spectrum of poor responses to therapy in patients with hypoxic tumours, and there is no effective way to identify those who will respond the worst. Thus, the development of classification tools intended to identify patients harbouring tumours at high risk of recurrence would be extremely beneficial for monitoring patient outcome after receiving treatment.

Our results indicate that hypoxia can regulate the expression of piRNAs which, in turn, may aid in the prediction of prognosis for patients with hypoxic tumours. Specifically, we found that: 1) piRNA expression patterns are different between hypoxic and non-hypoxic human tumours derived from a variety of anatomical sites; 2) hypoxia-mediated regulation of piRNAs occurs *in vitro*, resembling patterns observed in human tumours; and 3) a hypoxia-regulated piRNA expression signature can identify hypoxic tumours at higher risk of recurrence in HNSC, LUSC, and LUAD patients.

We deduced the oxygenation status of tumour samples processed by TCGA using a previously characterized and validated hypoxia gene expression signature from Winter *et al*.^41,42^. Using this method, tumours from all tissue types were classified into hypoxic or non-hypoxic groups, except for ovarian and kidney tissues. While this lack of clear classification between hypoxic and non-hypoxic ovarian cancer samples was not anticipated, it was expected in renal cell carcinomas because >75% of sporadic RCCs contain mutated or lost VHL^49^. Loss of VHL function mimics hypoxia-mediated stabilization of HIF-1α, which then dimerizes with HIF-1β to form HIF-1 and induce transcription. Constitutively activated HIF-1 in renal tumours, in part attributed to mutated VHL, upregulates genes involved in the hypoxic response regardless of local cellular oxygen tensions. Accordingly, we have previously found that piRNA expression is remarkably homogeneous across RCC patient samples^18^, suggesting piRNA expression patterns in RCC may be related to VHL status in these tumours. Thus, we sought to directly assess the ability of VHL to affect piRNA expression in a non-renal setting. To do this, we silenced VHL in a lung cancer cell line that had robust upregulation of piRNAs in hypoxia (Fig. 3). We observed increased expression of two different piRNAs when VHL was knocked down in A549 cells, and piRNA expression was decreased to control levels by simultaneous knockdown of HIF-1α (Fig. 4B,C). These data indicate that the VHL-mediated induction of DQ590404 and DQ596992 piRNAs is HIF-1α dependent, and suggest that the induction of piRNAs in hypoxic cells more generally may also be regulated by HIF-1.

In our analysis, we identified 40 piRNAs that are associated with hypoxia in patient tumours and regulated by hypoxia in cell models of the same tissue type. Interestingly, we found that most of the hypoxia-regulated piRNA species (36 out of 40; 90%) were upregulated in hypoxic compared to normoxic conditions, which is consistent with previous observations in a glioblastoma cell lines^50^. This phenomenon is opposite to what is observed in miRNA, where hypoxia mainly induces a reduction in expression levels by modulating their biogenesis^10^. Since piRNAs are generated through a Dicer-independent pathway, it is expected that the effect of low-oxygen tension will be different on piRNAs compared to miRNAs. Hypoxia-mediated regulation of piRNAs has also been described in breast cancer cell lines in a HIF dependent manner, although the absence of HIF binding sites in the vicinity of encoding loci suggest that are unlikely to be directly regulated by HIF^51^. Overall, our results provide novel mechanistic insights involving a VHL-mediated stabilization of HIF1a regulating the expression of a subset of piRNAs, and also highlight the importance of considering hypoxia-mediated regulation of piRNA expression in a tissue-specific manner.

Previous work has shown individual piRNAs that correlate with clinical features in different tumour types^28^. Also, multipiRNA signatures have been used to stratify patients by prognosis^39,46^, although the presence of some hypoxia-regulated piRNAs in these published piRNA signatures suggest potential identification of hypoxic tumours that are expected to have poorer prognosis from the bulk patient populations. Our PiSco signature enables stratification of patients with the most aggressive hypoxic tumours into low or high risk of recurrence, suggesting that analysis of hypoxia-regulated piRNA expression can be used as a predictive tool to prioritize outcome monitoring after treatment, even in patients with early-stage hypoxic lung adenocarcinoma (Fig. 5).

In conclusion, this proof-of-principle study reveals the influence of the tumour microenvironment on DNA-level regulatory mechanisms with important implications for predicting recurrence in patients with hypoxic tumours. Furthermore, we find that hypoxia-regulated piRNA expression can be altered by VHL status *in vitro*. Our data encourage further exploration of hypoxia-regulated piRNAs as clinical tools for evaluating the likelihood of tumour recurrence, and to identify patients that would most benefit from adjuvant therapies and/or therapies designed to target hypoxic tumour cells.

## Electronic supplementary material

Supplementary Figures

Table S1

Table S2
