# Supplementary material for: Non-coding RNAs predict recurrence-free survival of patients with hypoxic tumours
Source: Sci Rep. 2018 Jan 9;8:152. doi: 10.1038/s41598-017-18462-z (PMC5760628; doi:10.1038/s41598-017-18462-z)
Supplement: Supplementary file 1 — Supplementary Figures [file 41598_2017_18462_MOESM1_ESM.pdf]

Non-coding RNAs predict recurrence-free survival of patients with hypoxic tumours

Authors: Victor D. Martinez[1, †,*], Natalie S. Firmino[1, †], Erin A. Marshall[1, †], Kevin W. Ng[1], Brennan J Wadsworth[1], Christine Anderson[1], Wan L. Lam[1], Kevin L. Bennewith[1]

Affiliations: [1]Department of Integrative Oncology, British Columbia Cancer Agency, Vancouver, B.C. Canada V5Z 1L3.

*Corresponding author. Email: vmartinez@bccrc.ca

†These authors contributed equally to this work

Figure S1

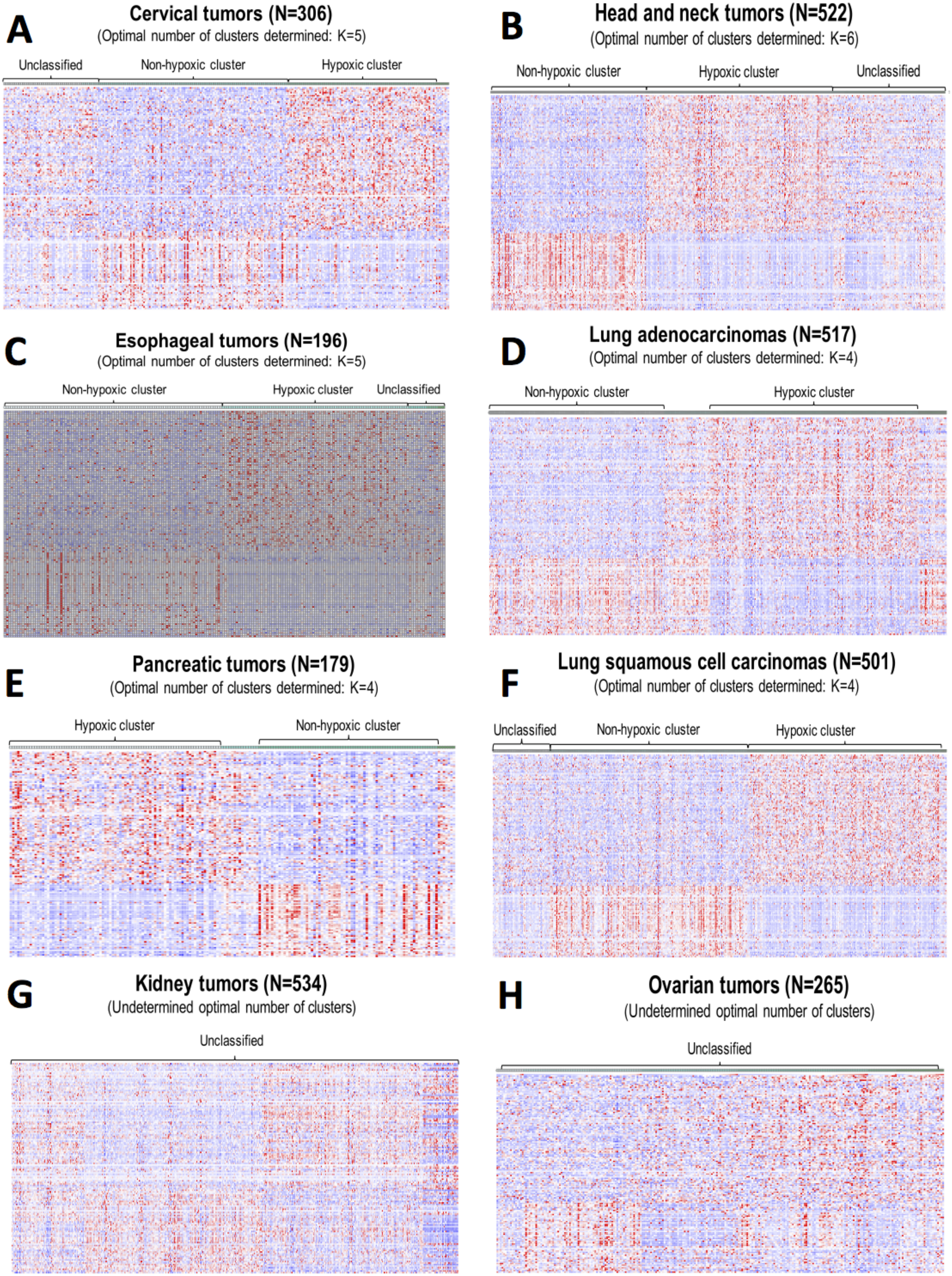
A
Cervical tumors (N=306)
(Optimal number of clusters determined: K=5)
Unclassified
Non-hypoxic cluster
Hypoxic cluster
B
Head and neck tumors (N=522)
(Optimal number of clusters determined: K=6)
Non-hypoxic cluster
Hypoxic cluster
Unclassified
C
Esophageal tumors (N=196)
(Optimal number of clusters determined: K=5)
Non-hypoxic cluster
Hypoxic cluster
Unclassified
D
Lung adenocarcinomas (N=517)
(Optimal number of clusters determined: K=4)
Non-hypoxic cluster
Hypoxic cluster
E
Pancreatic tumors (N=179)
(Optimal number of clusters determined: K=4)
Hypoxic cluster
Non-hypoxic cluster
F
Lung squamous cell carcinomas (N=501)
(Optimal number of clusters determined: K=4)
Unclassified
Non-hypoxic cluster
Hypoxic cluster
G
Kidney tumors (N=534)
(Undetermined optimal number of clusters)
Unclassified
H
Ovarian tumors (N=265)
(Undetermined optimal number of clusters)
Unclassified

Figure S2

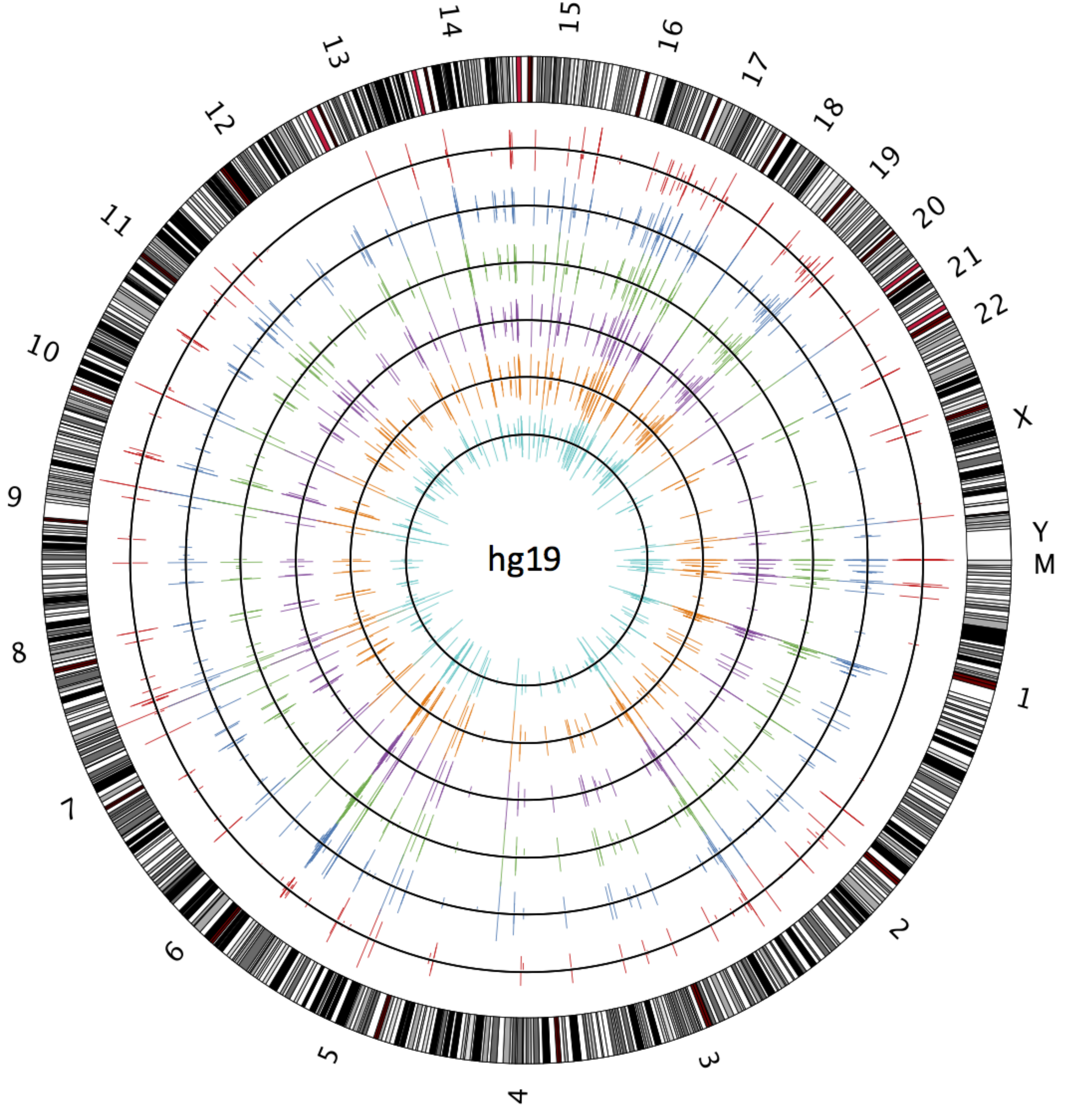

hg19
1
2
3
4
5
6
7
8
9
10
11
12
13
14
15
16
17
18
19
20
21
22
X
Y
M

Figure S3

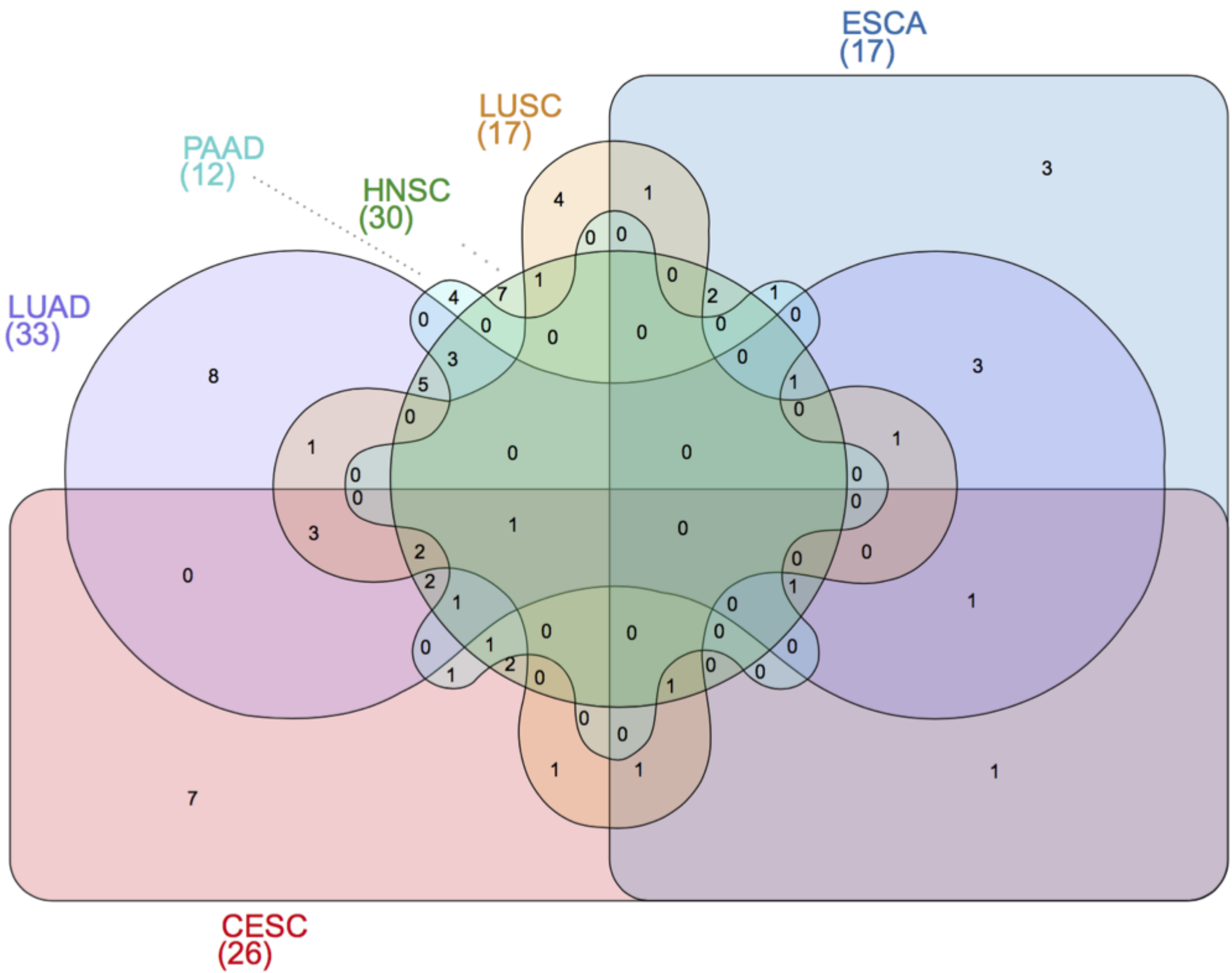
ESCA
(17)
LUSC
(17)
PAAD
(12)
HNSC
(30)
LUAD
(33)
CESC
(26)
